# ﻿Two new species of *Trichoglossum* (Geoglossaceae, Ascomycota) from south Mexico

**DOI:** 10.3897/mycokeys.92.83928

**Published:** 2022-08-30

**Authors:** Javier Isaac de la Fuente, Jesús García-Jiménez, Tania Raymundo, Daniyal Gohar, Mohammad Bahram, Marcos Sánchez-Flores, Ricardo Valenzuela, Juan P. Pinzón

**Affiliations:** 1 Colegio de Postgraduados, Km 36.5 Montecillo, Texcoco, 56230, Estado de México, Mexico Colegio de postgraduados Texcoco Mexico; 2 Tecnológico Nacional de México. Instituto Tecnológico de Ciudad Victoria. Blvd. Emilio Portes Gil #1301Pte, 87010, Ciudad Victoria, Tamaulipas, Mexico Tecnológico Nacional de México. Instituto Tecnológico de Ciudad Victoria Ciudad Victoria Mexico; 3 Instituto Politécnico Nacional, Escuela Nacional de Ciencias Biológicas, Departamento de Botánica, Laboratorio de Micología, 11340, Cd. Mx., Mexicos Instituto Politécnico Nacional, Escuela Nacional de Ciencias Biológicas Mexico Mexico; 4 Institute of Ecology and Earth Sciences, University of Tartu, Lai 40, 51005, Tartu, Estonia University of Tartu Tartu Estonia; 5 Department of Ecology, Swedish University of Agricultural Sciences, Ullsväg 16, 75651 Uppsala, Sweden Swedish University of Agricultural Sciences Uppsala Sweden; 6 Departamento de Botánica, Facultad de Medicina Veterinaria y Zootecnia, Universidad Autónoma de Yucatán, carretera Mérida-Xmatkuil km 15.5, 97100, Mérida, Yucatán, Mexico Universidad Autónoma de Yucatán Mérida Mexico

**Keywords:** Earth tongues, Geoglossomycetes, phylogeny, Quintana Roo, Tropical Ascomycetes

## Abstract

Two new species of *Trichoglossum* are described from south Mexico based on morphological and molecular evidence. *Trichoglossumcaespitosum* is characterized by the caespitose ascomata, rough and coiled paraphyses and the ascospores with 9–11 septa. *Trichoglossumtropicale* is characterized by the capitate ascomata, clavate and straight paraphyses and the ascospores with 10–12 septa. Both species grow in the tropical forests of the Yucatán peninsula. Here we provide descriptions and photographs for these species, together with a phylogenetic analyses based on the DNA sequences of nuc rDNA (ITS region and 28S gene) and a comparative table for the species known for America.

## ﻿Introduction

The members of the genus *Trichoglossum* Boud. are characterized by club-like ascomata, usually with dark brown to black hues and acuminate setae covering both fertile and sterile parts of the ascomata, septate paraphyses, asci with 4 to 8 spores, and filiform, septate, brown ascospores. The genus is saprotrophic but is also present at the roots of plants as endophytic fungi (Rinaldi et al. 2008; Tedersoo et al. 2010; Wang et al. 2011; Hustad et al. 2013). They have a worldwide distribution in tropical and temperate forests (Mains 1954; Beug et al. 2014). Although the genus has been the focus of many phylogenetic studies, several species lack molecular data, which obstruct a better understanding of its phylogenetic relationships (Schoch et al. 2009; Ekanayaka et al. 2017). So far, 22 species of *Trichoglossum* are currently accepted (Ekanayaka et al. 2017; Lee et al. 2021; Chakraborty et al. 2022; Dasgupta et al. 2022; Index Fungorum, accessed May 2022).

In Mexico, five taxa of *Trichoglossum* have been recorded, mainly in temperate forest and even urban gardens: *T.hirsutum* (Pers.) Boud., T.hirsutumvar.hirsutum (Pers.) Boud., T.hirsutumvar.heterosporum Mains, *T.variabile* (E.J. Durand) Nanff., *T.velutipes* (Peck) E.J. Durand, and *T.walteri* (Berk.) E.J. Durand (Ramírez-López and Villegas-Ríos 2007; Raymundo et al. 2016). Most of the *Trichoglossum* collections have been made in Central Mexico, followed by a few collections from southern Mexico (Díaz-Barriga 1988; Ramírez-López and Villegas-Ríos 2007; Rodríguez-Alcántar et al. 2021; Valenzuela et al. 2021), so far, no *Trichoglossum* species has been recorded from the Yucatán Peninsula. In recent years, we have conducted several mycological explorations in southern Mexico, mainly in the state of Quintana Roo. During those explorations, several collections of *Trichoglossum* species were made, which resulted in identification of two new species. The aim of this study is to describe *Trichoglossumcaespitosum* and *T.tropicale* supported by molecular and morphological characters. Further, a comparative table is provided for the species known for America.

## ﻿Methods

### ﻿Sampling data

**Figure 1.**
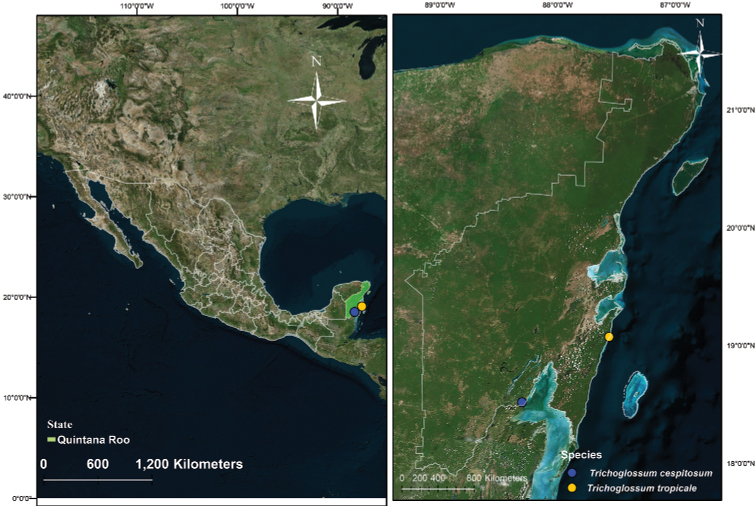
Map showing the collecting sites.

Sampling of macrofungi was carried out in the Mexican state of Quintana Roo, in the Yucatán Peninsula (Fig. 1). The representative vegetation at the sampling sites was an urban garden with *Manilkarazapota* and an ecotone between a lowland forest and mangrove forest with *Yuccaelephantipes*, *Metopiumbrownei*, *Gymnopodiumfloribundum*, *Conocarpuserectus*, and *Byrsonimacrassifolia*. Hand cuts were made in dried specimens and mounted with KOH 5%; Melzer reagent was used to observe amyloid asci apex. At least 30 ascospores, asci, and paraphyses were measured to obtain ranges. The material was deposited at mycological collections of Instituto Politécnico Nacional (**ENCB**), .Universidad Autónoma de Yucatán (**UADY**), and Instituto Tecnológico de Ciudad Victoria (**ITCV**).

### ﻿DNA extraction, amplification, and sequencing

Total DNA was extracted from silica-gel dried ascomata of the collected samples (specimens JF-208-ITCV and JF-526-ITCV), following a proteinase K protocol (Thermo Fisher Scientific, Waltham, MA, USA) according to Põldmaa et al. (2019). Samples were incubated in 100 µl of lysis buffer [0.8 M Tris-HCl, 0.2 M (NH_4_)_2_SO_4_, 0.2% w/v Tween-20; Solis BioDyne, Tartu, Estonia] and 2.5 μl of proteinase K at 56 °C for 24 h, following by 15 min at 98 °C, and finally centrifuged for 2 min at 8000 rpm. Region nuc 28S rDNA (LSU) was amplified with the primers LR0R (Cubeta et al. 1991) and LR7 or LR5 (Vilgalys and Hester 1990), using 5× HOT FIREPol. Blend Master Mix (Solis BioDyne, Tartu, Estonia). The PCR protocol followed consisted of 35 cycles of 95 °C for 40 s, 55 °C for 1 min, and 72 °C for 1 min. ITS region nuc rDNA The Internal Transcribed Spacer (ITS) was amplified with the primer ITS5-ITS4 (White et al. 1990). The PCR protocol consisted of 35 cycles of 95 °C for 35 s, 58 °C for 1 min, and 72 °C for 2 min. The amplification program was run as follows: denaturalization at 95 °C for 4 min, 35 cycles of denaturalization at 95 °C for 1 min, annealing at 58 °C for 1 min, polymerization at 72 °C for 2 min, and final elongation at 72 °C for 10 min. Purification of the PCR products was performed with Exo-Sap enzymes (Sigma, St. Louis, Missouri) and sequenced at the Estonian Biocentre (Tartu, Estonia). Sequences were assembled in BIOEDIT 7.2.5 (Hall 1999), and compared against the sequences deposited in NCBI’s database with a blastn analysis, using megablast (highly similar sequences). The sequences generated were deposited at NCBI GenBank.

### ﻿Phylogenetic analyses

The taxa selection for the phylogenetic analysis was based on the available sequences of ITS and 28S of *Trichoglossum* in NCBI GenBank database considering the analysis of Geoglossomycetes by Hustad et al. (2013). All main clades within the class are represented, including all representatives of the *Trichoglossum* clade. Additionally, other specimens with ITS and 28S sequences available were included, such as *Hemileucoglossumpusillum* (GB: MF353090/NG_060706), *Leucoglossumleucosporum* (GB: KP272114/KP272115), and an uncultured Geoglossaceae (GB: D1273321/DQ273452). The molecular matrix was aligned using the MUSCLE algorithm (Edgar 2004), and variable and parsimony informative characters were calculated in MEGA 11 (Tamura et al. 2021); the DNA substitution model used in the phylogenetic analyses was selected following the Akaike Information Criterion (AIC) in MODELTEST 2.1.7 (Darriba et al. 2012).

A Bayesian inference analysis (BI) was performed in MrBayes 3.2.6 (Ronquist and Huelsenbeck 2003; Ronquist et al. 2012) of the concatenated matrix of ITS and 28S regions, which consisted of 1408 positions (703 of ITS and 705 of LSU), with 532 variable sites (37.8%) and 340 parsimony informative sites. (24.1%). The following parameters were set: substitution model GTR+I+G for both markers, two independent runs of 10 million generations, sampling every 1000 generations with one cold chain and three hot chains and the remaining parameters used as default. The substitution rates, character state frequency, gamma shape and proportion of invariable sites were unlinked for both partitions.

Additionally, a Maximum Likelihood (ML) analysis was carried out using the GTR+I+G substitution model and bootstrap (BS) based on 100 replicates using MEGA 11 (Tamura et al. 2021). The trees produced through the BI and ML analyses were visualized and edited with FigTree v.1.4.3 (Rambaut 2016).

## ﻿Results

### ﻿Phylogenetic analyses

For 28S, the blastn analysis of the collected specimens showed high levels of similarity (over 93%) with accessions of several genera within Geoglossales as well as with uncultured fungi from environmental DNA sequencing. The specimen JF-208-ITCV showed a maximum score with *Trichoglossumrasum* (GB: KY457227) (100% query cover, 98.69% identity), and the specimen JF-526-ITCV with *Trichoglossumhirsutum* (GB: KC222146) (100% query cover and 95.65% identity). For ITS, the sequence retrieved for the specimen JF-208-ITS was 384 bp in length, spanning the 18S gene (SSU) only (partial), so it could not be used for the phylogenetic analysis; however, the blastn analysis showed that the most similar sequences belong to the family Geoglossaceae: *Gluginoglossum* sp. (GB: OM672838), and two specimens of *Trichoglossum* sp. (OM474029, OM672708), all with 78% of query cover and 78.86% of identity. For the specimen JF-526-ITCV the sequence was 528 bp and contained ITS1, 5.8S and ITS2; the most similar sequences in the blastn analysis were several specimens of *Trichoglossum* sp. from New Zealand, showing a query cover of 100% and identity over 99.24% (OM987287, OL653059, MH578528, OL653016, HQ222864); the most similar sequence of an identified specimen at the specific level was from *T.hirsutum*, also from New Zealand (query cover 99%, identity 98.67%).

**Figure 2.**
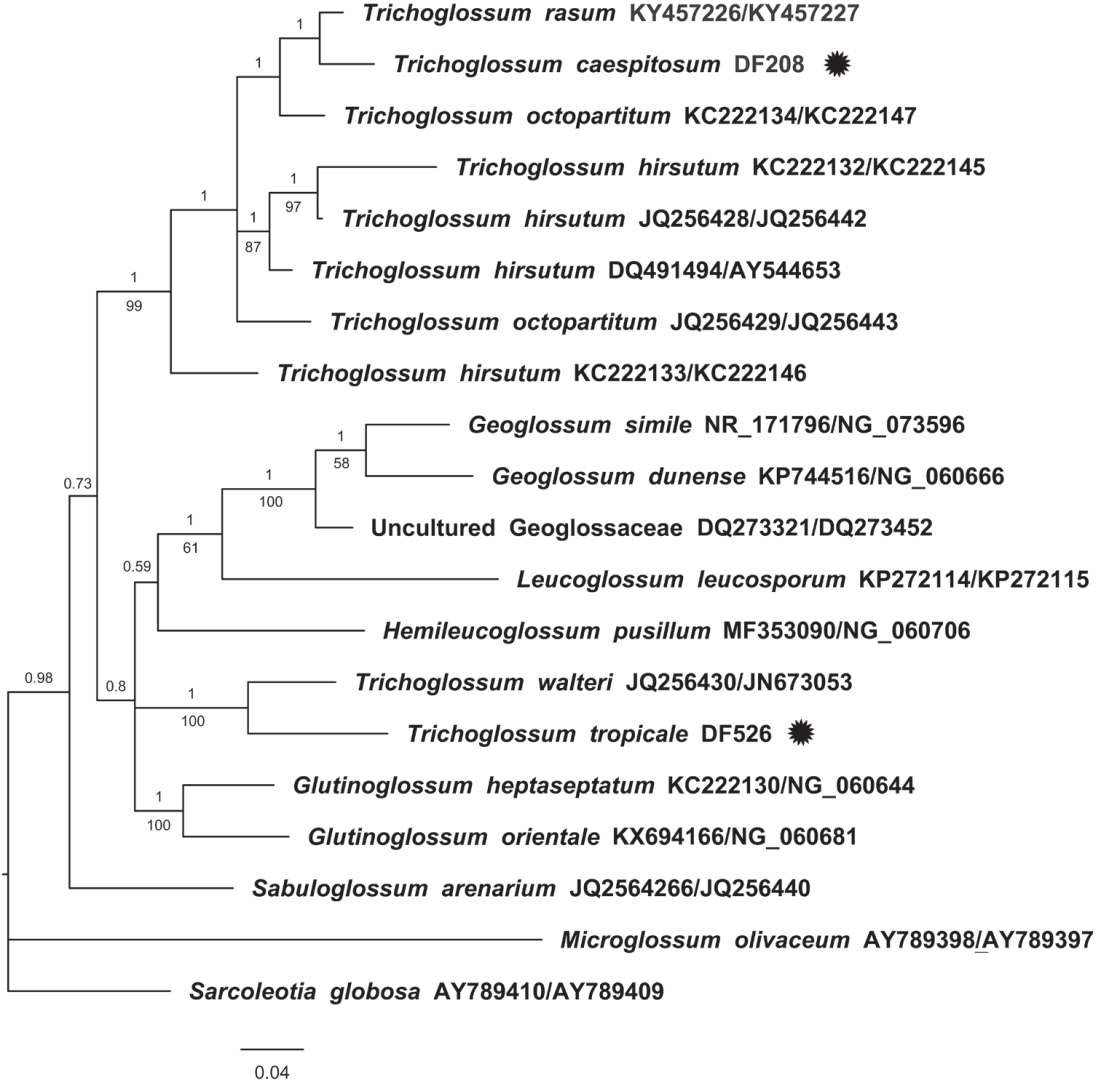
Majority rule consensus tree produced by a Bayesian Inference analysis of the concatenated matrix of nuc rDNA ITS1-5.8S-ITS2 and 28S (LSU) showing the phylogenetic position of *Trichoglossumcaespitosum* (JF-208-ITCV) and *T.tropicale* (JF-525-ITCV) (marked with stars) within the Geoglossomycetes. GenBank accession numbers are indicated (ITS/28S). The posterior probabilities are shown above the branches, and bootstrap values (above 50) from a Maximum Likelihood analysis, below.

The majority rule consensus tree produced by the BI analysis (Fig. 2) shows a moderately well supported clade (PP = 0.96) confirmed by all specimens of Geoglossomycetes, except for *Sarcoleotiaglobosa*, which is collapsed at the base of the tree, together with *Microglossumolivaceum* (Leotiomycetes). Within the clade of Geoglossomycetes, *Sabuloglossumarenarium* is sister to the rest of the species (with a low posterior probability of 0.73), and two clades are identified, one formed by species of *Glutinoglossum*, *Hemileucoglossum*, *Leucoglossum*, *Geoglossum*, an uncultured Geoglossaceae, and the specimen JF-526-ITCV sister to *Trichoglossumwalteri*, with a high support (PP = 1, BS = 100). The second clade is well supported (PP = 1, BS = 99), and it is composed exclusively with the rest of the species of *Trichoglossum* in which we find the specimen JF-208-ITCV, which is sister to *T.rasum* with a high posterior probability (PP = 1). Our phylogenetic analysis of *T.hirsutum* specimens suggests that the species is polyphyletic in its current circumscription. The topology of the ML tree (not shown) shows some incongruences with the BI majority rule tree, such as the position of *Hemileucoglossumpusillum* and of the specimen JF-208-ITCV, which is here sister to the clade of *T.hirsutum*-*T.octopartitum*-*T.rasum* but with low support (BS = 68). The remaining relationships were congruent with those obtained with the Bayesian Inference analysis.

### ﻿Taxonomy

#### ﻿ Trichoglossum caespitosum

Taxon classificationFungiGeoglossalesGeoglossaceae

de la Fuente, J. García & Raymundo sp. nov.

04656CAC-FCEE-5A36-BE95-10207C85F9E2

 843008

Figs 3A–E , 5A–D

##### Holotype.

Mexico. Quintana Roo: Othón P. Blanco Municipality, Chetumal, alt. 8 m, 18°31'N, 88°18'W, 01 December 2015, de la Fuente JF-208-ITCV; Isotype UADY 04867. GenBank: OM727118.

**Figure 3.**
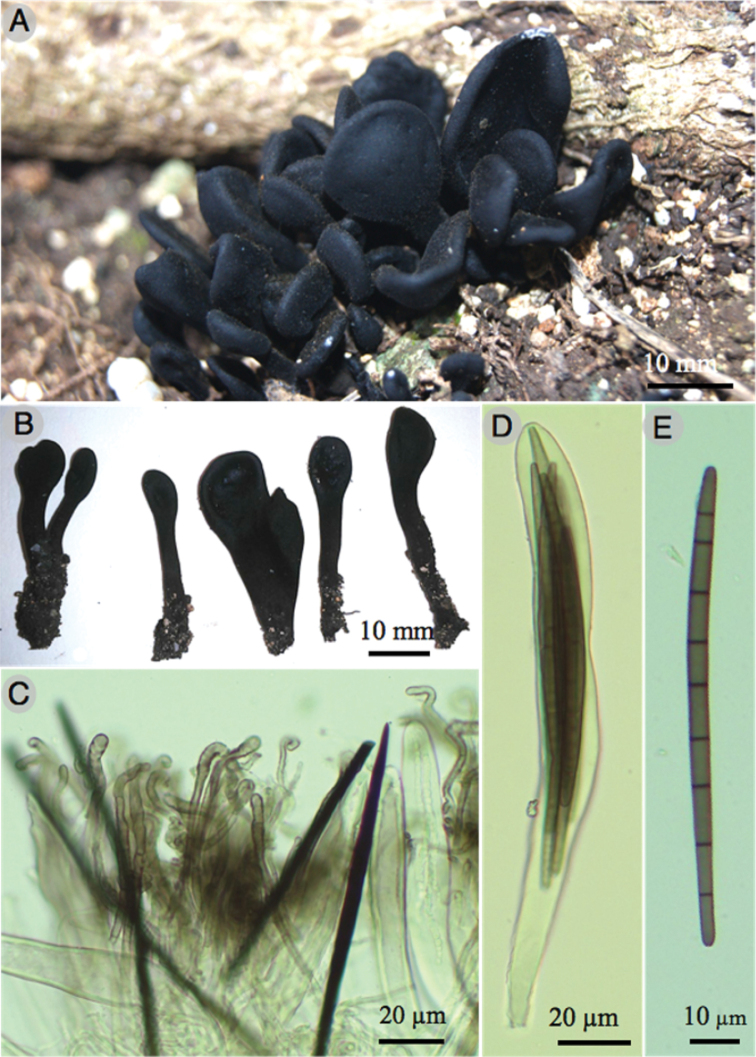
*Trichoglossumcaespitosum* (Holotype) **A, B** ascomata **C** paraphyses, setae, and asci **D** detail of asci **E** ascospores. All microstructures are mounted in KOH.

##### Diagnosis.

*Trichoglossumcaespitosum* is characterized by the unique combination of characters: Caespitose habit, paraphyses with rugose and coiled tips, and the ascospores of 119–127 × 5–7 µm with 9–11 septa.

##### Etymology.

Named *caespitosum* in reference to the caespitose habit.

##### Description.

***Ascomata*** black, 18–30 × 4–10 mm, clavate to spathulate, stipitate, erect, caespitose, with compressed ascogenous portion of 3–7 mm long, 0.5–1 mm thick, glossoid, ellipsoidal, flattened, sometimes curved, black, hirsute with brownish to black setae projecting from the hymenium; ***stipe*** 1–33 mm long, up to 6 mm thick, cylindrical, solid, black to dark brown, hirsute.

***Setae*** 250–300 × 5–9 µm, septate, smooth, straight, dark brown to black. ***Paraphyses*** filamentous, septate, with rugose, with wide, coiled to clavate terminal cells of 16–28 × 3–8 µm. ***Asci*** 183–221 × 16–20 µm, cylindrical to clavate, rounded at apex, short-pedicellate at the base, hyaline, thin walled, octosporic, inamyloid. ***Ascospores*** 119–127 × 5–7 µm, filiform, mostly 9–11 septate, slightly curved, hyaline when young, brown to olivaceous when mature, narrowed and rounded at both ends, thin walled, smooth.

##### Distribution.

Known from the Mexican state of Quintana Roo, growing on soil under *Manilkarazapota* in urban vegetation.

##### Notes.

This species differs from other species by the caespitose ascomata, paraphyses with rugose and coiled tips, and the ascospores of 119–127 × 5–7 µm with 9–11 septa. *Trichoglossumrasum* Pat. is morphologically similar by the octosporic asci, clavate spathulate ascoma with dark brown hues, and the tropical distribution but it differs in the bigger ascomata (10–60 mm), smaller setae (200–250 × 5–12 µm), larger ascospores (100–140 × 5–8 µm) with 3–7 septa (Mains 1954). *Trichoglossumoctopartitum* Mains can also be found in the Yucatan peninsula biotic province and shows similar ascoma and ascospore size; nevertheless, the ascomata is never caespitose ascomata and shows 7-septate ascospores (Mains 1954; Ekanayaka et al. 2017). *Trichoglossumconfusum* E.J. Durand has also similar ascoma size but differs in the smaller ascospores (57–75 × 5–7 µm) with 3–7 septa (Mains 1954).

#### ﻿ Trichoglossum tropicale

Taxon classificationFungiGeoglossalesGeoglossaceae

de la Fuente, Sánchez-Flores & Raymundo sp. nov.

AF5F3BA3-B67B-53F0-AA62-FED8A6CA0A60

 843009

Figs 4A–F , 5E–H.

##### Holotype.

Mexico. Quintana Roo: Othón P. Blanco Municipality, Pulticub town, alt. 6 m, 19°04'N, 87°33'W, 04 February 2021, de la Fuente JF-526-ITCV, Isotype ENCB 140350. GenBank: OM727119.

**Figure 4.**
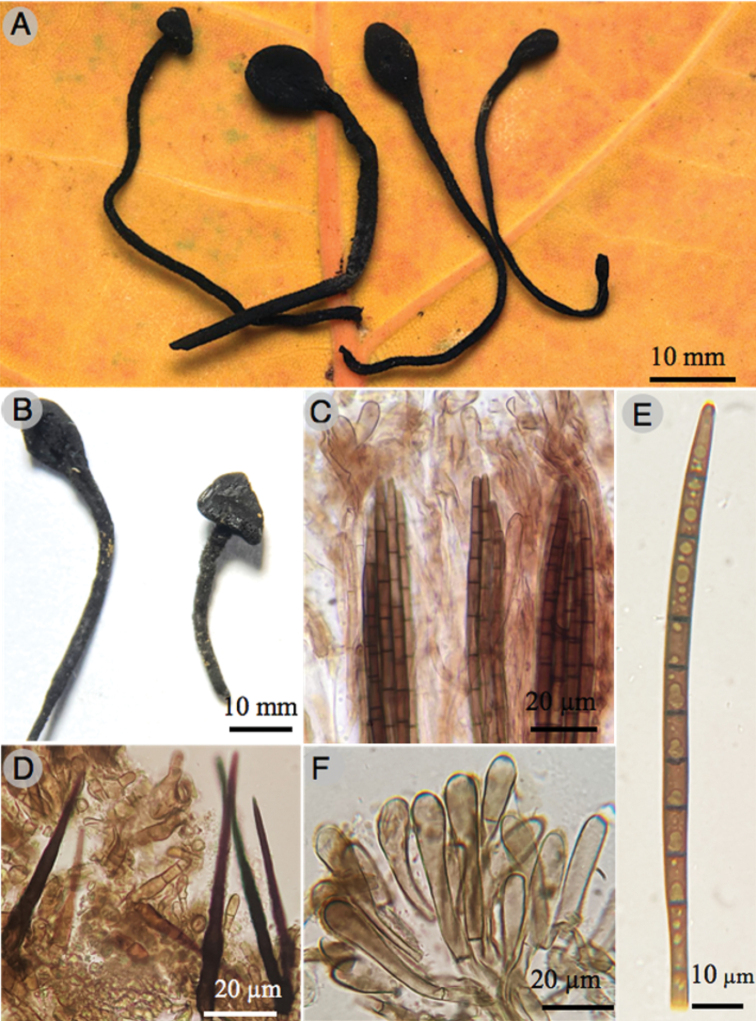
*Trichoglossumtropicale* (Holotype) **A, B** ascomata **C** asci **D** detail of setae **E** ascospore **F** detail of paraphyses. All microstructures are mounted in KOH.

##### Diagnosis.

*Trichoglossumtropicale* is characterized by the combination of characteristics: capitate ascomata, straight paraphyses with bulbose tips, and the ascospores of 122–132 × 5–5.5 µm with 10–12 septa.

**Figure 5.**
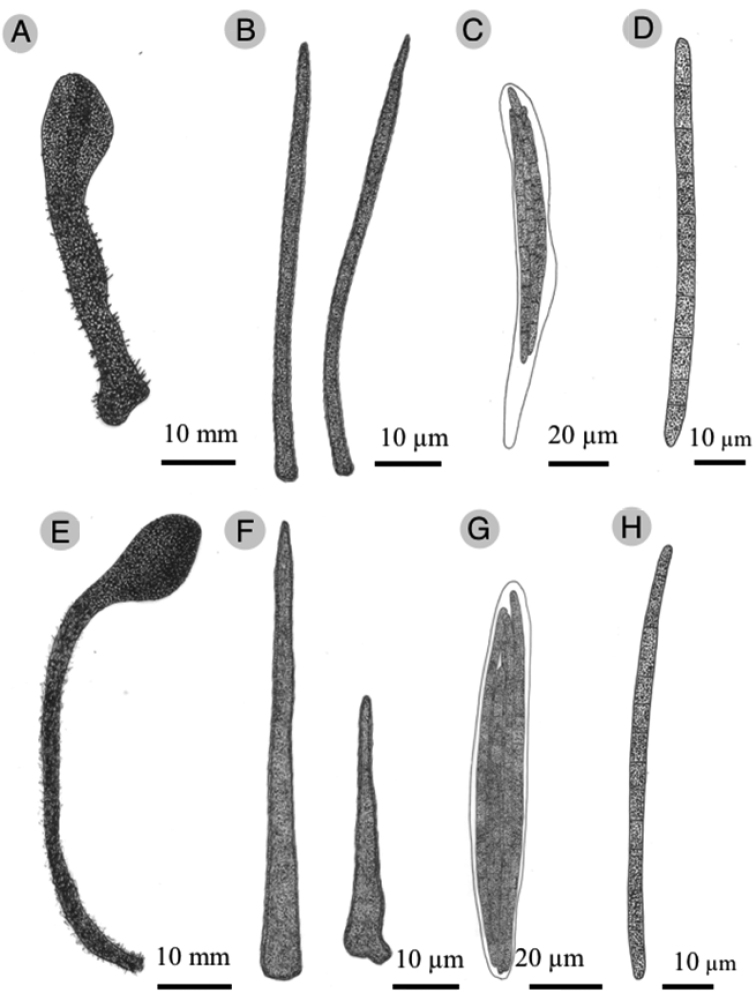
New *Trichoglossum* from Mexico. *Trichoglossumcaespitosum***A** ascomata **B** setae **C** ascus **D** ascospore. *Trichoglossumtropicale***E** ascomata **F** setae **G** ascus **H** ascospore.

##### Etymology.

Named *tropicale* in reference to the tropical occurrence.

##### Description.

***Ascomata*** black, 15–30 × 2–4 mm, clavate to capitate, stipitate, erect, solitary to gregarious with compressed ascogenous portion of 2–4 mm long, 1–2 mm thick, glossoid, ellipsoidal, flattened, sometimes curved, black, without visible setae. ***Stipe*** 10–20 mm long, up to 1 mm thick, cylindrical, solid, black to dark brown, hirsute.

***Setae*** 98–200 × 5.5–6 µm, septate, smooth, straight, dark brown to black. ***Paraphyses*** filamentous, septate, with capitate to bulbous terminal cells of 8–46 × 6–10 µm. ***Asci*** 155–180 × 16–18 µm, cylindrical to clavate, rounded at apex, short-pedicellate at base, hyaline, thin walled, octosporic, inamyloid. ***Ascospores*** 122–132 × 5–5.5 µm, filiform, mostly 10–12 septate, slightly curved, hyaline when young, brown to olivaceous when mature, narrowed and rounded at both ends, thin walled, smooth.

##### Distribution.

Known from the Mexican state of Quintana Roo, growing scattered on soil under *Birsonymacrassifolia*.

##### Notes.

This new species differs from other *Trichoglossum* species by the combination of characteristics: ascomata with inconspicuous setae (98–200 × 5.5–6 µm), straight paraphyses with bulbose tips, and the ascospores of 122–132 × 5–5.5 µm with 10–12 septa. *Trichoglossumtropicale* is phylogenetically close to *T.walteri* but that species has ascospores of 60–125 × 5–6 μm with 7 septa and paraphyses curved to circinate (Mains 1954). A similar species is *T.hirsutum* due to the capitate ascomata, setae size (up to 225 µm long); differs in the thicker setae and larger ascospores (90–150 × 5–7 µm) with 15 septa (Beug et al. 2014). *Trichoglossumvelutipes* has similar ascospore septation, but it has four-spored asci and bigger ascospores (110–145 × 6–7 µm) with 7–11 septa (Ekanayaka et al. 2017). *Trichoglossumvariabile* has similar number of septa, but differs in the presence of four-spored asci, smaller setae (69–183 × 7.6–12 µm), and bigger ascospores (80–150 × 6 µm) with 9–14 septa (Mains 1954; Beug et al. 2014).

## ﻿Discussion

The results of the phylogenetic analyses are concordant with those of Hustad et al. (2013), recovering the Geoglossomycetes clade (except for *Sarcoleotiaglobosa*, collapsed at the base), as well as the *Geoglossum*, *Glutinoglossum*, and *Trichoglossum* clades. The inclusion of *Trichoglossumwalteri* and *T.tropicale* in this clade make the genus non-monophyletic. Both species, *T.walteri* and *T.tropicale*, form a small clade close to *Glutinoglossum*, nevertheless both species have setae, an absent feature within *Glutinoglossum*. Based on morphology and phylogenetic data (data set nuc rDNA), we describe and propose *Trichoglossumcaespitosum* and *T.tropicale* as new species that inhabit tropical vegetations and associate with *Manilkarazapota* and *Birsonymacrassifolia* respectively in the Yucatán Peninsula.

*Trichoglossumcaespitosum* is very close morphologically and phylogenetically to *T.rasum* (KY457226; KY457227), but differs because of its ascospores of 100–140 × 5–8 μm, 3–9 septa, bulbous and curved tips paraphyses; this species was described from New Caledonia (Patouillard 1909) and later it was cited from Bermuda, Cuba, Panama (Mains 1954) and India (Prabhugaonkar and Pratibha 2017). *Trichoglossumoctopartitum* is phylogenetically related but differs mainly in the non-caespitose ascomata and the 7-septate ascospores (Mains 1954). According to our phylogenetic study, *T.caespitosum* is related to *T.hirsutum*. This species has been recorded in America, Asia and Europe; nevertheless, this could be a species complex or polyphyletic group and needs further detailed study (Hustad et al. 2013). The main differences between *T.caespitosum* and *T.hirsutum* are the caespitose ascomata of the new species and the number of septa per ascospore. Whereas the ascospores of *T.caespitosum* show 9–11 septa, the ascospores of *T.hirsutum* generally show 15 septa; ascospores with fewer than 15 septa are rare according to Mains (1954). *Trichoglossumtropicale* is phylogenetically close to *T.walteri* (JQ256443) from North Carolina but differs in that it has spores with a size of 90–100 × 4–5.5 μm and paraphyses curved to circinate, besides, the type specimen of *T.walteri* is from Australia, but it was cited from Brazil, Jamaica, and the United States of America (Durand 1908; Mains 1954).

According to the blastn analysis of the ITS region, several sequences from New Zealand are close to the one obtained from *T.tropicale*. Nevertheless, those species are not described yet. Ekanayaka et al. (2017) gave a morphological description of a specimen from China that they referred to as *T.cf.octopartitum*. ITS sequences provided by Hustad et al. (2013) revealed that the species is also located in North America (being Belize the type locality) and Europe as well. Microscopical differences of the American species of *Trichoglossum* are presented in Table 1.

**Table 1.** Comparison of the species of *Tricholglossum* from America (according ^1^Mains 1954; ^2^Chacón and Guzmán 1983; ^3^Ramírez-López and Villegas-Ríos 2007, and ^4^this work).

Species	Asci (L = length × W = width)	Ascospores (L = length × W = width)
***T.caespitosum* ^4^**	184–220 × 16–20 μm, octosporic	120–128 × 5–7 μm, 9–11 septate
***T.confusum* ^1^**	150–200 × 12–16 µm, octosporic	45–75 × 5–6 μm, 7 septate
***T.farlowii* ^1^**	150–180 × 15–20 µm, octosporic	57–75 × 5–7 μm, 3–5 septate
***T.hirsutum* ^1^**	180–275 × 18–25 μm, octosporic	80–170 × 5–7 µm, 15 septate
**T.hirsutumvar.hirsutum ^1,2^**	180–250 × 14–16 (21.5) µm, octosporic	(80) 110–140 (170) × 5–7 µm, mostly 15 septate
**T.hirsutumvar.longisporum ^1^**	225–275 × 20–22 µm, octosporic	(120) 133–180 (195) × 6–7 µm, 15 septate
**T.hirsutumvar.irregulare ^1^**	No data	(90) 100–150 (165) × 5–7 µm, 15 septate, rarely 16–17
**T.hirsutumvar.heterosporum ^1,3^**	175–200 × 17–20 µm, octosporic	(95) 120–150 (160) × 5–6 (7) µm, less than 15 septate
**T.hirsutumvar.multiseptatum ^1^**	210–225 × 20–25 µm, octosporic	(145) 160–195 (210) × 6 µm, 12–22 septate
***T.octopartitum* ^1^**	175–200 × 18–20 μm, octosporic	(80) 100–120 (150) × 4–5.5 μm, 7–9 septate
***T.rasum* ^1^**	200–225 × 16–24 μm, octosporic	(50) 100–140 (175) × 5–8 μm, 3–9 septate
***T.tetrasporum* ^1^**	175–200 × 20–25 μm, tetrasporic	(110) 125–145 (150) × 6–7 µm, 0–17 septate
***T.tropicale* ^4^**	155–180 × 16–18 µm, octosporic	122–132 × 5–5.5 µm, 10–12 septate
***T.variabile* ^1^**	150–200 × 18–20 µm, octosporic	(80-) 110–130 (-150) × 4.5–6 µm, 4–16 septate
***T.velutipes* ^1^**	180–200 × 16–20 µm, tetrasporic	(90) 110–145 (160) × 6–7 µm, 7–13 septate
***T.walteri* ^1^**	165–200 × 15–18 μm, octosporic	(60) 72–100 (125) × 5–6 μm, 7 septate

## Supplementary Material

XML Treatment for Trichoglossum caespitosum

XML Treatment for Trichoglossum tropicale
